# Evaluation and treatment for ovotesticular disorder of sex development (OT-DSD) - experience based on a Chinese series

**DOI:** 10.1186/s12894-017-0212-8

**Published:** 2017-03-28

**Authors:** Yu Mao, Shaoji Chen, Ru Wang, Xuejun Wang, Daorui Qin, Yunman Tang

**Affiliations:** 1 Department of Pediatric Surgery of Children’s Medical Center, Sichuan Academy of Medical Sciences & Sichuan Provincial People’s Hospital, Chengdu, China; 2 Department of Burn and Plastic Surgery, West China Hospital of Sichuan University, Chengdu, China

**Keywords:** Ovotestis, Hypospadias, Urethroplasty, Disorder of sex development

## Abstract

**Background:**

The aim of this study is to review and present the clinical features and process of evaluation and treatment for OT-DSD in a single center in recent years in China.

**Methods:**

Sixteen patients with OT-DSD during the past 4 years underwent the evaluation and treatment in a single center. The clinical characteristics and outcomes of surgery were analyzed.

**Results:**

The surgical age ranged from 17 months to 66 months with a mean age of 20 months, and the mean follow-up was 30 months (4 months to 56 months). The presentation in 11 patients was ambiguous genitalia, and the rest 5 patients were suspected to have DSD in preoperative examination before hypospadias repair. The karyotypes were 46, XX in 11 patients, 46, XX/46, XY in 3, 46, XX/47, XXY in 1, and 46, XY in 1. Initial reared sex was male in 14 patients, female in 1, and undetermined in 1. After surgery, genders were reassigned in 3 patients, while 15 patients were raised as male with testicular tissue left. Only 1 patient with ovarian tissue left was raised as female. Repair was completed in 11 males and 1 female, and stage I urethroplasty was done in 4 males. No further surgery to remove the gonads was needed for inconsonance of gender assignment. No gonadal tumors were detected.

**Conclusions:**

OT-DSD is a rare and complex deformity with few systematic reports in China. It’s important to establish a regular algorithm for evaluation and treatment of OT-DSD.

## Background

The nomenclature ovotesticular disorder of sex development (OT-DSD) has replaced the obsolete one, true hermaphroditism, since 2006 [1]. It is defined by the presence of testicular tissue with well-developed seminiferous tubules and ovarian tissue with primordial follicles in the same individual. These tissues may be co-existent in the same gonad (ovotestis) or independently the ovary on one side and the testicle on the other). The incidence is rare, accounting for nearly 3 to 10% of DSD cases [2]. The external genitalia show variable phenotypes, ranging from a normal male to a normal female presentation. However, ambiguous genitalia is the most common manifestation as noted in 90% of the cases [3]. The deformed genitalia compromises psychosocial as well as physiological health of the patients and their families. In China, data are in great need on the clinical features, assessing modalities, surgical procedures and outcomes, and the prognosis in the involved patients. We share the features of OT-DSD in our recent series.

## Methods

**Fig. 1:**
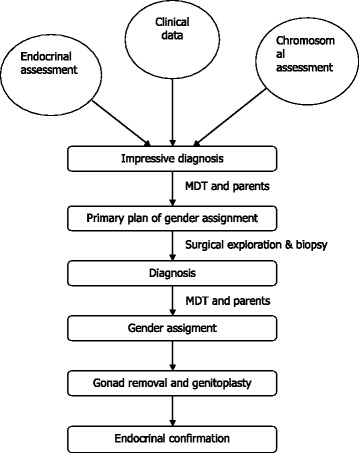
The procedure for diagnosis and management of OT-DSD patients in our hospital

From September 2011 to December 2015, 16 patients with OT-DSD were evaluated and treated in our hospital. As shown in Fig. 1, the procedure for diagnosis and management of OT-DSD was step by step, as first collection of clinical data, second chromosomal and endocrinal assessment, third multidisciplinary team (MDT) consultation, then surgical & histopathological confirmation of gonads nature with gender assignment, gonad(s) removal and genital plastic surgery in accordance with assigned gender. The final diagnosis depended on gonadal histopathology.

Clinical data included age, rearing gender, medical history, physical examination, imaging and screening of associated anomalies. Prader grading of the external genitalia and palpation of gonads are of essential importance in physical examination. Although the diagnostic accuracy of the internal genital organs was unsatisfactory via ultrasonography and MRI [4, 5], we still used these imaging modalities to characterize urogenital system before surgery.

Karyotype analysis was detected from the peripheral blood in all patients.

Although the diagnosis depended on histopathological findings, we utilized biochemical examination and human chorionicgonadotropin (HCG) test to distinguish OT-DSD from other DSDs before surgery. Before HCG test, serum testosterone (T), luteinizing hormone (LH), follicle stimulation hormone (FSH), anti-müllerian hormone (AMH), InhibinB, 17-hydroxyprogesterone (17-OHP), androstenedione, and sex hormone binding globulin (SHBG) were detected. Consecutive HCG injection, a dose of 100 units/kg/day, was indicated for 3 days. At the 4th day, T, LH, FSH, androstenedione, SHBG, and dihydrotestosterone (DHT) were detected again. The T concentration after HCG stimulation of higher than 200 ng/dl was defined as a normal response, 100 to 200 ng/dl as a borderline response, lower than 100 ng/dl as a poor response.

Patient’s data were submitted to MDT, including pediatric endocrinologist, andrologist, gynecologist, pediatric urologist, geneticists, pathologist, psychologist, and medical ethics committee, for consultation. Then the parents were involved in the discussion of possible gender assignment.

**Fig. 2:**
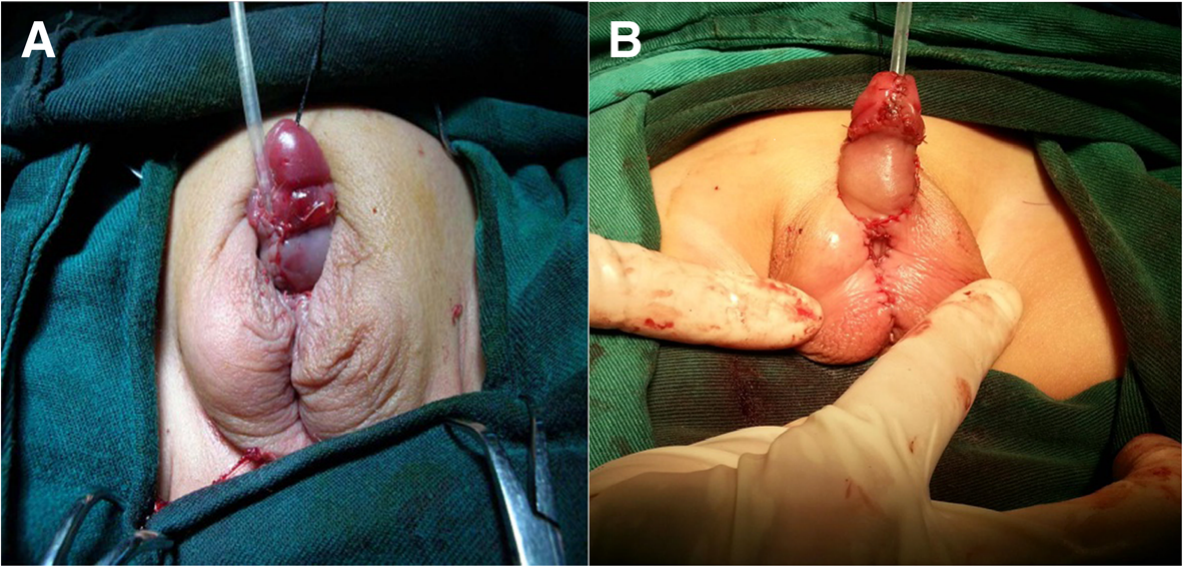
The appearance after the first stage of two-stage techniques we routinely used in urethroplasty. **a** Plate reconstruction with two-stage tubularizationurethroplasty. After the first stage, the new meatus is in the shaft of the phallus. **b** Ducketturethroplasty-urethrostomy by stages. After the first stage, the urethroplasty is finished except a fistula

Surgery included 3 parts. A cystoscopy with a 3Fr catheter was used to measure the length of urogenital sinus, water-filled vagina and the distance from the bladder neck to the vagina meatus, as well as to identify the existence of cervical orifice at the end of the vagina. The second step was gonadal detection, biopsy and selected removal. Laparoscopy was applied to confirm the components of intraperitoneal gonads and internal genital ducts combining with punctural biopsy or excisional biopsy. Parents would make final decision of gender assignment based on the results of exploration before gonad removal during the operation. Occasionally, Müllerian duct structures were removed via laparoscopy. On the basis of dominant gonads and the consensus of gender assignment, only one type of gonad was reserved, and the other was resected. The third step was genitoplasty. Clitoroplasty, vaginoplasty, and labioplasty were carried out in female gender. According to the length of urogenital sinus, we used partial urogenital mobilization (PUM) or flap vaginoplasty for vaginoplasty. Orthoplasty, urethroplasty and scrotoplasty were carried out in male gender. Plate reconstruction with two-stage tubularization urethroplasty [6] and Duckett urethroplasty-urethrostomy by stages [7] were the two-stage techniques we routinely used in severe hypospadias repairs (Fig. 2).

One month after surgery, for patient reassigned as female, human menopausal gonadotropins (HMG) test [8] with a daily intramuscular dose of 150 IU on 3 consecutive days was indicated to verify no residuary testicle tissue. While for patients reassigned as male, HCG test was carried out to confirm no residuary ovarian tissue. All the patients were followed up at 1, 3, 6, 12 months after surgery and every year thereafter.

All procedures used in the study confirmed to the tenets of the Declaration of Helsinki. The Ethics Committee of Sichuan Academy of Medical Sciences & Sichuan Provincial People’s Hospital approved the protocols used. All participants have known to participate in the study. Written informed consents were obtained from all participants.

## Results

As shown in Table 1, the age ranged from 17 months to 66 months with a mean age of 20 months, and the mean follow-up was 30 months (4 months to 56 months). Eleven patients came to our hospital for ambiguous genitalia and 5 patients were referred to our institution for hypospadias repair. No relevant family history or associated anomalies were noted. According to Prader classification, stage II to V were recorded with stage III accounting for 62.5% (10 out of 16). Müllerian remnants were detected in 8 patients before surgery via ultrasonography or MRI.

**Table 1:** The characteristic of these OT-DSD patients

No	Karyotype	Prader grade	MR imaging	T post HCG (ng/dl)	L gonand		R gonad		L duct	R duct	UVB (cm)	Uterus	Gender assignment	Complication
					Position	Tissue	Position	Tissue						
1	46,XX	III	+	367.5	Ing	OT	Ing	OT	Tube	Tube	3.5/4.5/2.5	+	M	No
2	46,XY	III	-	213.3	Abd	OT	Abd	OT	Tube	Tube	2/5/4	-	M	S
3	46,XX/46,XY	II	+	379.9	Abd	O	Ing	OT	Tube	Tube	3.5/5/4.5	+	M	No
4	46,XX	IV	-	416.2	Scr	OT	Abd	O	Tube	Tube	4/6/3	+	M	No
5	46,XX	III	+	209.1	Ing	OT	Ing	OT	Tube	Tube	2.5/4.5/3.5	+	M	F
6	46,XX	III	+	541.1	Scr	OT	Scr	T	Tube	Vas	1.5/6.5/5.5	+	M	No
7	46,XX	III	+	254.4	Scr	OT	Scr	T	Tube	Vas	1/6.5/5	+	M	No
8	46,XX/46,XY	III	-	322.6	Scr	OT	Abd	O	Vas	Tube	2/5.5/4.5	+	U → M	No
9	46,XX/47,XXY	III	-	186.4	Ing	T	Abd	OT	Vas	Tube	2.5/4.5/3.5	+	M	No
10	46,XX	II	+	462.8	Scr	OT	Scr	OT	Vas	Vas	2.5/5/4	+	M	No
11	46,XX/46,XY	III	-	275.4	Ing	OT	Ing	OT	Tube	Tube	3/5.5/4	+	M	^a^
12	46,XX	II	+	302.2	Abd	O	Scr	T	Tube	Vas	3.5/6/3.5	+	M	^a^
13	46,XX	III	-	166.3	Abd	O	Ing	OT	Tube	Tube	1/5.5/5.5	+	F → M	^a^
14	46,XX	III	+	532.6	Scr	OT	Scr	OT	Tube	Vas	2/5/4.5	+	M	^a^
15	46,XX	II	+	178.6	Abd	O	Scr	OT	Tube	Tube	3/5/3.5	+	M → F	No
16	46,XX	V	-	346.2	Scr	OT	Scr	OT	Vas	Vas	5/4/3.5	-	M	^a^

*MR* Müllerian remnant, *L* Left, *R* Right, *UVB* Length of urogenital sinus/ water-filled vagina/ the location of the vaginal confluence to the bladder neck, *Ing* Inguinal cana, *Abd* Abdominal cavity, *Scr* Scrotum, *OT* Ovotestis, *O* Ovary, *T* Testicle, *Tube* Fallopian tube, *Vas* Vas deferens, *M* Male, *F* Female, *U* Undetermined sex, *F* Fistula, *S* Urethral stricture, ^a^Didn’t finished the second stage

With HCG stimulation, a normal response was noted in 13 patients, and the rest 3 patients showed a borderline response. Follow-up in puberty is important for the 3 borderline response patients. The other endocrinal indexes were normal or meaningless for considering other DSD.

In this group of patients, the karyotypes were 46, XX in 11 patients (68.75%), 46, XX/46, XY in 3 (18.75%), a rare karyotype 46, XX/47, XXY in 1 (6.25%) and 46, XY in 1 (6.25%).

The most common gonad was ovotestis (68.75%), followed with ovaries (18.75%), and the least common was testis (12.5%). Ovotestis was easily noticed in the scrotum (50%), subsequently in the inguinal canal (36.4%), then in the abdominal cavity (13.6%). All the 6 ovaries were discovered in the abdominal cavity. Three testes were found in the scrotum and the other one in the inguinal canal. Adjacent to the ovotestis, internal genital duct was verified as vas deferens on 6 lateralities via biopsy and as fallopian tube in 16. Bilateral ovotestis was the most frequent pattern of the gonads as seen in 7 patients. An ovotestis on one side and an ovary on the other side was noted in 5 patients, while an ovotestis on one side and a testis on the other side was noted in 3 patients. In only 1 patient a testis on one side and an ovary on the other was noted.

The vagina was found out in 16 patients with cystoscopy. At the end of the vagina, a cervical orifice was noted in 14 patients. The mean length of urogenital sinus (U), i.e., the length from confluence of the vagina and the urethra to the urethral meatus was 2.7 cm. The mean length of water-filled vagina (V) was 5.3 cm. The mean distance from the vaginal confluence to the bladder neck (B) was 4.1 cm.In the Table 1, we use the abbreviation UVB to describe the three distance.

During surgery, gonadal gender was determined via frozen section biopsy. In all the patients, the frozen pathological outcomes fell into anticipated possibilities of preoperative MDT consultation. Gender assignment was then decided accordingly. Initial reared sex was male in 14 patients, female in 1, and undetermined in 1. The gender was reassigned in 1 patient who was primarily reared as male, 1 as female, and the previously undetermined 1 was assigned as male. All the gonads and adjacent ducts in abdominal cavity were subjected to bipolar punctural biopsy via laparoscopy. All the ovotestes presented in a bipolar fashion. Staged urethroplasty was indicated in all the patients assigned as male. Plate reconstruction with two-stage tubularization urethroplasty (PRTU) was used in 10 patients. Stage II urethroplasty was achieved in 8 patients with urethrocutaneous fistula noted in 1 and urethral stricture in 1 as postoperative complications. Two patients just finished the first stage without complication. Duckett urethroplasty-urethrotomy by stages was adopted in 5 patients. Three patients finished the fistula repair and 2 patients just finished the first stage of Duckett urethroplasty with a fistula left. No complications were found in the 5 cases (Table 1). Müllerian ducts were removed in 4 patients and left intact in 11. Clitoroplasty and partial urogenital mobilization (PUM) were carried out in only 1 patient who was assigned as female. No complications were noticed in this patient during the follow-up.

**Fig. 3:**
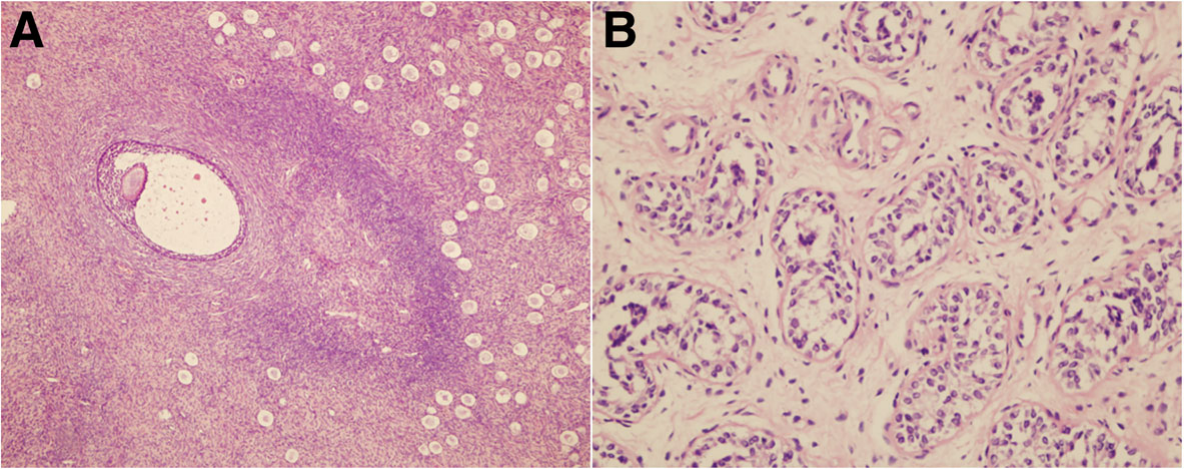
Ovotestis in OT-DSD. **a** The ovarian compartment has numerous primordial and growing follicles containing primary oocytes within the ovarian cortical stroma. **b** The testicular compartment shows solid tubules filled with immature Sertoli cells and germ cells. The testicular interstitium contains immature Leydig cells

Histopathology was used as gold standard of final diagnosis. All the immediate outcomes of frozen section during operation were accordant with that of paraffin section after operation (Fig. 3). The testicular tissues presented numerous solid seminiferous tubules filled with immature Sertoli cells and a few primitive germ cells. The testicular interstitium contained undifferentiated spindle cells that were immature Leydig cells. In the ovarian tissue, numerous primordial follicles and a few primary and antral follicles were found in the outer cortex.

In the first month of the follow-up, HCG/HMG test was completed with no evidence of residual inconsonant gonad. No gonadal tumors were noted during the follow-up.

## Discussion

Ethicists and patient support groups advocated that the genital surgery should not be warranted until the patient was able to understand the informed consent, instead of repair in infancy period [9]. However, as the appearance of bisexsual phenotype and continuous anxiety of parents call for the management, it is rational and in a degree mandatory to initiate the evaluation at an early age. Ambiguous or undermasculinized genitalia of OT-DSD is easy to detect, while decision making is difficult when the genital appearance seems to be normal female or otherwise normal hypospadiac male [10]. Physical examination for the gonads is important. In 5 patients out of this series, OT-DSD was suspected as a tough nodule adjacent to the testicle or bipolar asymmetrical texture of the testicle was palpated in physical examination for hypospadias preoperative evaluation. The Imaging only revealed 8 patients with internal genital ducts, however, the vagina was found out in all the 16 patients with cystoscopy. Imaging modalities to characterize urogenital system before surgery is also inaccurate.

The karyotype showed geographic variation. It was interesting that our data showed a similar pattern of karyotype in this Chinese series as that reported in Europe and North America, in contrast to that in Japan [11]. In Japan the 46, XY karyotype is more common than that in other countries [12]. The mosaicism of Klinefelter syndrome with 46,XX/47,XXY in OT-DSD is very rare with less than 10 cases reported worldwide [13]. Our patient 9 is 2 years and 5 months old with this karyotype. His manifestation as listed in Table 1 showed no difference to that with other karyotypes of OT-DSD. Though sex-determining region on the Y chromosome (SRY) is an important gene in testicular development, the implication of SRY presence in OT-DSD remains indeterminate. SRY detection is not indicated in our institution as a routine.

**Fig. 4:**
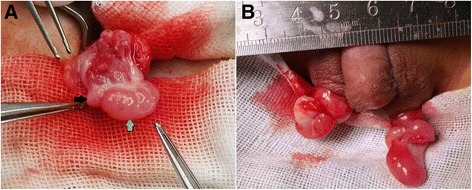
Intraoperative findings of ovotestes. **a** Ovarian portion (*black* arrow) is firm and *yellow* in an upper pole, whereas testicular portion (*green* arrow) is soft and pink in a lower pole. There is a distinct line of demarcation between the two portions. **b** The demarcation between the two portions is not obvious in the 16th patient

Ovotestis is the most common gonad in OT-DSD as reported in most articles [13, 14]. Wiersma and Ramdial [14] evaluated the gonads from 111 patients with OT-DSD in South Africa. They proposed three distinct patterns of gonad, namely the admixed pattern that was central core containing stroma and a mixture of ovarian and testicular tissue (50%), the compartmentalized pattern that was ovarian tissue in upper pole with lower pole of testicular tissue encapsulated by mantle of ovarian tissue (39%), and the bipolar pattern that was strict polar distribution of testicular and ovarian tissue (11%). In our patients, most ovotestes were of bipolar type, which was end to end fashion (Fig. 4a). Ovarian tissue located on the upper pole, was cerinous, rigid, and smaller than testicular tissue. On the contrary, the testicular tissue located on the lower pole, was buff, softer and larger than ovarian tissue. Demarcation was significant between the two parts. The demarcation between ovarian tissue and testicular tissue in the 16th patient was not obvious on gross view (Fig. 4b). While the different texture made it evident to distinguish the boundary. This might be the type assigned as compartmentalized by Wiersma and Ramdial, except for that ovarian tissue was not found out in the mantle with microscopy. In OT-DSD, either testicular part of ovotestis or isolated testis presents maldeveloped microscopic features. On the contrary, either ovarian part of ovotestis or isolated ovary presents well-developed microstructures. In microscopy, the presence of numerous primordial follicles containing primary oocytes with or without maturing follicles is considered well developed ovarian tissue, which making a definitive diagnosis of OT-DSD. Menstruation is expected in 50% cases based on well-developed ovarian tissue [15]. The negative feedback effect of ovarian steroids suppressing gonadotropins results in tubular atrophy, poor germ cell development, Leydig cell hyperplasia, and sclerosis that finally causes infertility of the testicular tissue. As the individual gets older, the damage on the testicle in ovotesitular patient would deteriorate [15]. This is another reason why we indicate gender assignment, especially for those who with male dominance, in infancy. If the pathologists have misgivings about the nature of the gonads in the frozen section, the next step should be postponed awaiting the outcomes of hematoxylin-eosin staining.

Being the most common form of OT-DSD, ovotestis should be screened out during preoperative physical examination according to the typical morphological features. Internal genital ducts adjacent to the ovotestis are usually difficult to identify with naked eyes, and frozen biopsy is warranted in surgery.

On the basis of PVE classification system [16], we designed UVB measurement to assess the location of vagina confluence and the length of urogenital sinus and vagina. The critical factor in the vaginoplasty is not the length of the common sinus but rather the distance from the bladder neck to the location of vagina confluence. The former index is very useful in the surgical planning of vaginoplasty. Partial urogenital mobilization (PUM) or flap vaginoplasty is indicated based on these evaluations.

Our multidisciplinary team and the families took part in the gender assignment. Prader grading, karyotype, nature and function of gonads, values of UVB, presence of Müllerian ducts, psychological assessment and living environment are critical factors involved in the gender assignment. However it is difficult when the decision from the MDT and that from the parents are conflicting. According to Chinese tradition that the boys carry on the family lines, most conservative families prefer male to female in OT-DSD after thorough evaluation, as well as worry about the catastrophic effect of gender reassignment on the whole family. Even with Prader II genitalia, possibility of fertility for female assignment, which lead the MDT suggesting female more appropriate, most parents still insist on male as final gender. The MDT would try to fully inform the parents with professional evaluation, but the final decision is made by the parents. All the three families accepted gender reassignment chose to move to a remote place for a new life.

Laparoscopy is widely utilized in the exploration of gonads and resection of Müllerian ducts. Resection of Müllerian duct derivatives as routine several years ago is not recommended in patients without any symptom nowadays. However, Farikullah reported 3 to 8% incidence of malignancy in Müllerian remnants [17]. Informing the parents with this incidence of malignancy, we were surprised that removal of the müllerian ducts was required in all the latest 4 asymptomatic patients for the risk of malignancy. Of course, if the gender couldn’t be decided during the operation or the final gender was female, The Müllerian remnants must be retained. Bipolar and multisite punctural biopsy of gonads in abdominal cavity with laparoscopy is minimally invasive and these gonadal biopsies are enough to achieve histopathological evaluation for diagnosis. Repair of severe hypospadias is challenging and the complication rate is always high. During the recent years in our institution, plate reconstruction with two-stage tubularization urethroplasty [6] and Duckett urethroplasty-urethrotomy by stages [7] are the main two-stage techniques we routinely indicate in primary severe hypospadias repair. The complications of the two technique in primary severe hypospadias repair in our hospital are 16.7 and 9.43%, respectively [6, 7]. Both the two techniques are suitable for urethroplasty in OT-DSD.

The low incidence of Y chromosome in karyotype and young age of the patients induced no gonadal tumor to be detected. The incidence of gonadal tumors is approximately 3% in 46, XY and 46, XX/46, XY OT-DSD, though rare in 46, XX OT-DSD. Both gonadoblastoma and dysgerminoma have been described [18]. HMG/HCG test is an important content in the follow-up. The residual gonad inconsistent with rearing sex will result in bisexual phenotype in puberty.

## Conclusions

OT-DSD is a rare and complex malformation with lots of typical features. Standardized procedure of evaluation and treatment for OT-DSD is very important. MDT consultation might guarantee the high efficiency and accuracy in evaluation and treatment. Chinese parents prefer male to female when they face to the gender reassignment.

## Abbreviations

17-OHP: 17-hydroxyprogesterone
AMH: Anti-müllerian hormone
DHT: Dihydrotestosterone
DSD: Disorder of sex development
FSH: Follicle stimulation hormone
HCG: Human chorionic gonadotropin
HMG: Human menopausal gonadotropins
LH: Luteinizing hormone
MDT: Multidisciplinary team
MRI: Magnetic Resonance Imaging
OT-DSD: Ovotesticular disorder of sex development
PUM: Partial urogenital mobilization
SHBG: Sex hormone binding globulin
T: Serum testosterone
